# Enumeration of Somatic and F-RNA Phages as an Indicator of Fecal Contamination in Potable Water from Rural Areas of the North West Province

**DOI:** 10.3390/pathogens4030503

**Published:** 2015-07-01

**Authors:** Keitumetse Idah Nkwe, Collins Njie Ateba, Nomathamsanqa Patricia Sithebe, Cornelius Carlos Bezuidenhout

**Affiliations:** 1 Department of Biological Sciences, School of Environmental and Health Sciences, North West University, Mafikeng Campus, Mmabatho, Mafikeng 2735, South Africa; E-Mails: atebacollins1@hotmail.com (C.N.-A.); thami.sithebe@nwu.ac.za (N.P.S.); 2 Food Security and Safety Niche Area, Faculty of Agriculture, Science and Technology, North-West University, Mmabatho, Mafikeng 2735, South Africa; 3 Unit for Environmental Science and Management, Faculty of Natural Science, North-West University, Potchefstroom Campus, Potchefstroom 2531, South Africa; E-Mail: Carlos.Bezuidenhout@nwu.ac.za

**Keywords:** somatic phage, F-RNA phage, rural water supply, groundwater, potable water contamination

## Abstract

Bacteriophages are regarded as enteric viral indicators in faecally contaminated water systems and may indicate the presence of human viral pollution. They are relatively resistant to inactivation by natural and treatment processes. In this study, the presence of somatic coliphages and F-RNA coliphages was investigated in potable water from rural areas in the North West province. Water samples were aseptically collected from boreholes and tap water from some rural communities in the North West Province. Physical parameters of the water, such as the temperature, pH and turbidity, were measured before sample collection. Double-agar layer assay was performed using ISO, (1995, 2000) standard methods. Bottled water was used as a negative control and the strains фX174 and MS2 as positive controls. Of the 16 water samples collected, 15 were positive for somatic bacteriophages while F-RNA coliphages were detected in only two samples. Amongst the positive samples 189 and three plaque forming units were obtained for both somatic and F-RNA coliphages, respectively. No coliphage was detected in water from Masamane tap 1. The rest of the samples obtained from various rural areas were positive and did not comply with national and international standards for potable water. This was a cause for concern and should be further investigated.

## 1. Introduction

In South Africa, the availability of safe and clean water is a serious problem particularly in rural areas in the North West Province. Individuals who live in areas use water directly from available sources without any treatment and, therefore, are exposed to a variety of water-related diseases [1,2,3,4,5,6,7].

The faecal coliform group of bacteria has been used as a water quality parameter and indicator of faecal pollution [8,9,10]. The presence of bacteria of faecal origin in water indicates that other intestinal pathogens could also be present in water. However, the absence of faecal indicator bacteria does not necessarily imply that pathogens are absent [11]. Furthermore, resistance of various pathogenic microorganisms to the water purification process is wide ranging and dependent on many factors. Some pathogenic organisms may survive the disinfection process and land into distribution systems. Moreover, some viruses and parasites are usually more resistant to the water treatment processes than the currently utilized bacterial indicators and therefore may be more reliable for applications that are involved in assessing water quality [12,13,14]. Against this backdrops, somatic coliphages [15,16,17], F-specific bacteriophages [18,19] and bacteriophages infecting *Bacteroides fragilis* [12,19,20] are currently used as suitable indicators of faecal pollution particularly in drinking water systems. These organisms also provide possible indications of the presence of human enteric viruses in water. Monitoring of all pathogenic viruses in water is very important for routine water quality assessment purposes [21]. For this reason, bacteriophages had been included as a valuable parameter for drinking water globally as well as in South Africa [22].

The purpose of this study was therefore, to determine the presence of two types of bacteriophages (somatic coliphages and F-RNA bacteriophages) in potable water sourced from the rural areas of the North West Province. F-RNA bacteriophages (ss RNA linear genome) are similar to Hepatitis A virus (HAV) and enterovirus but very different to rotaviruses (ds RNA fragmented genome) and are considered to be good indicators of the presence of enteric viruses in water that are not specific to humans. Somatic coliphages when compared to F-RNA bacteriophages have been found to multiply in the environment, which limits their use to a large extent [23,24,25].

## 2. Materials and Methods

### 2.1. Water Collection Methods

Potable water samples were collected from rural communities in the North West Province of South Africa. A total of 16 potable water samples were collected from both taps and boreholes. Physical parameters such as temperature, pH and turbidity were measured *in situ* using a Crison machine (Lasec) multimeter. Sample collection was done during the July 2013.

Water samples were collected in a 500 mL sterile whirl pack sample container and transported on ice in a dark container to the Microbiology laboratory in the Department of Biological Sciences, North West University–Mafikeng Campus for analysis. The water samples were analysed for the presence of bacteriophages within 24 h of collection. Water samples were stored at 4 °C before analysis.

### 2.2. Enumeration of Bacteriophages from Water Samples

#### 2.2.1. Enumeration of Somatic Coliphages

The isolation of somatic bacteriophages was performed using the double-layer agar plaque assay [26]. Briefly the following approach was used: Top agar consisting of 2.5 mL sterile liquefied tryptone yeast glucose-extract agar (TYGA) containing 1 mL nalidixic acid (Sigma, St. Louis, MO, USA) and 600 µL of calcium chloride (CaCl_2_; Sigma, St. Louis, MO, USA) was held at 50 °C in a water bath. This was added to a test tube containing 1 mL of the host (*E. coli* WG5) and 1 mL of the water sample. The absorbance of the host culture was measured at 600 nm at time 0 and after 2 h using a spectrophotometer (Merck Chemicals (Pty) Ltd., Modderfontein, South Africa). Competent cells with an OD of 0.4 to 0.5 were used in the bacteriophage isolation procedure. The liquified agar mixture was poured onto the bottom agar layer (TYGA) that had been pre-dispensed and allowed to set in a 90 mm Petri dish. This procedure was carried out in duplicate. The phage фX174 was used as a positive control strain. The top agar was allowed to solidify before the plates were inverted and incubated overnight at 37 °C. During analysis, one vial of *E. coli* WG5 host culture was thawed at room temperature and boosted by adding approximately 25 mL of sterile nutrient broth and incubated for 2 h, while shaking at 37 °C. A 5 mL of nutrient broth taken at time 0 was used as a blank reference.

#### 2.2.2. Enumeration of F-RNA Bacteriophages

The isolation of F-RNA bacteriophages was also performed using the double- layer agar plaque assay [27]. However, in this case *Salmonella typhimurium* 3 Nal^r^ (F′ 42 *lac*:Tn5)] WG49 (ATCC 700730) and bacteriophage MS2 were used as the host culture and control F-RNA phage respectively. Top agar (TYGA) contained 1 mL of calcium glucose, 400 µL nalidixic acid held at 50 °C in a water bath was added to the test tubes containing 1mL of the host culture (WG49) and 1mL of water sample. The rest of the procedure was carried out as previously described in Section 2.2.1.

## 3. Results

**Figure 1:**
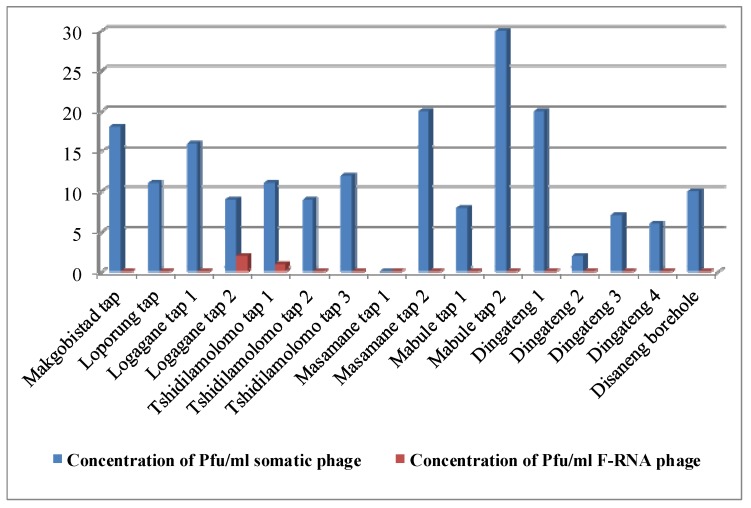
Areas and the concentration of viruses per milliliter of drinking water.

Table 1 shows the results of the physical properties that was obtained for the various potable water samples analysed while data for both somatic and F-RNA bacteriophages (pfu/100 mL) are shown in the Figure 1. The values obtained for physical parameters were compared to standard reference values on the SANS 241 (Table 1) drinking water quality guidelines [22] that were used by South African authorities to ascertain if the quality of water is in compliance with the appropriate drinking water standards.

**Table 1:** Physical properties of various potable water samples collected.

Areas SANS 241:2011	Turbidity (NTU) ≤ 5	pH ≥ 5 to ≤9.7	Temperature None
Makgobistad tap	141.2	7.59	18.0
Loporung tap	88.93	8.07	16.1
Logagane tap 1	68.19	7.21	26.8
Logagane 2	1.06	7.12	20.7
Tshidilamolomo tap 1	0.04	6.70	18.5
Tshidilamolomo tap 2	102.9	6.70	22.9
Tshidilamolomo tap 3	0.26	6.70	21.2
Masamane tap 1	0.46	7.78	27.3
Masamane tap 2	1.58	6.84	17.4
Mabule tap 1	1.01	7.50	24.4
Mabule tap 2	2.35	7.32	18.5
Dingateng 1	1.74	7.80	17.6
Dingateng 2	24.25	7.81	19.9
Dingateng 3	64.42	7.78	17.6
Dingateng 4	68.75	7.80	29.7
Disaneng borehole	107.7	7.08	24.1

NTU = Nephelometric turbidity unit.

Of the 16 water sources, 50% had NTU values greater than the maximum recommended values for drinking water and in some cases the values exceeded 100 NTUs. Water temperatures were above 16 °C. Despite the fact that there are no recommended temperature limits for drinking water, the values obtained in the present study were quite warm for the sampling period (July 2013). The pH values obtained for water samples ranged from 6.70–8.07 and these were within the recommended range for drinking water as indicated SANS 241 (2011) [22]. Despite this water from Tshidilamolomo, taps had pH values that were slightly acidic. One water sample from Loporung had a pH value of 8.07 and it is known that this may result to hardness in water. Unfortunately, hardness of the water was not determined.

Phage analysis data of the water samples are provided in Figure 1. From these results, it is evident that somatic bacteriophages were more prevalent in the water samples analyzed when compared to F-RNA bacteriophages. In general, somatic bacteriophages were detected in all except one of the water samples (Figure 1). On the contrary, F-RNA bacteriophages were detected only in two of the water samples analysed. The detection of large numbers (189) of plagues for somatic bacteriophages in 15 of the 16 potable water samples was a huge cause for concern and this indicated that these water samples did not comply with the standards set by SANS 241 [22].

## 4. Discussion

The present study was designed to determine the physicochemical quality of groundwater that is consumed by individuals in some rural communities in the North West Province, South Africa. A further objective was to determine the occurrence of both somatic and F-RNA bacteriophages in the samples analyzed. Results indicated that the pH values obtained for water samples were within the recommended range for drinking water as set by SANS [22]. There is no recommended temperature limits for drinking water set by SANS, but the values obtained in the present study were quite warm for the sampling period (July 2013).

Drinking water quality is a very important issue of concern world-wide and in semi-arid regions, such as South Africa, groundwater is the most important water resource [28]. This therefore implies that the quality of the water is of great concern. However, increasing human and industrial activities and the physical geographical conditions in a given area can result in complex chemical composition and high total dissolved solids (TDS) in the shallow groundwater bodies [29,30,31]. These factors can contribute to an increase in undesirable characteristics in water resulting in concentrations that may exceed both national and international drinking water standards [29,30,31]. All the water samples analysed were untreated groundwater obtained from boreholes. Generally, groundwater has always been considered safe and free from contamination. However, during the rainy season, total dissolved solids were higher than recommended limits in 50% of the water samples. Although the reason for the elevated TDS values cannot be clearly explained, it is suggested that differences in biological activities may account for these fluctuations.

There is no specific health implication associated pH content of drinking water except when values are extremely high, but the detection of both somatic and F-RNA bacteriophages indicated the presence of faecal bacteria contaminants [32]. Moreover, it has been reported that microbial contaminants in groundwater such as viruses, bacteria, and parasitic protozoa pose a significant human health problem to consumers when drinking water supplies are untreated or inadequately treated [33]. In the study, the present study contaminated surface material may have percolated through the soil and transported human faecal wastes into the water bodies. The presence of pathogens is usually associated with high risks of waterborne diseases in consumers, and this is of huge public health concern. On the contrary, some enteric viruses including bacteriophages found in water, may also originate from non-human faecal material [34]. Considering that the presence of pathogens have enhanced risks on a classic group of debilitated subjects (very young, old, pregnant, and immuno-compromised individuals), there is need to implement specific measures aimed at reducing the risk of waterborne infections in growing and weaker populations especially in the rural communities from which the samples were collected.

The ability of bacteriophages to survive under unfavorable conditions is highly diversified. It is therefore documented that there are a number of external physical and chemical factors, such as temperature and pH that may influence the survival of and persistence of bacteriophages in environmental sources [35,36,37,38,39,40,41,42,43]. Temperature is a crucial factor for the survival of somatic and F-RNA bacteriophages in water and other environments such as the soil [35,40,41]. Temperature plays a major role in the attachment, penetration, multiplication and the length of the latent period of bacteriophages. Higher temperatures can also lengthen the duration of the latent stage [41]. It has been demonstrated that temperatures between 8 °C and 22 °C significantly result in the inactivation of F-RNA bacteriophages and faecal coliforms than somatic bacteriophages [35]. The temperature of water samples analysed in the present study was on average 21.3 °C, which ranged from 16.1 °C to 29 °C for individual samples. This may explain why more somatic than F-RNA bacteriophages were isolated in the study. The pH is also one of the factors affecting the survivability of the phage [37,39]. A previous study demonstrated the effects of temperature and pH on the inactivation rate for related somatic bacteriophages [36]. It was shown that the inactivation rate of those bacteriophages was low between pH values of 6 to 8 [36]. Another study demonstrated that pH and temperature are the main factors affecting the persistence of F+RNA bacteriophages [43]. The pH range of the present study was between 6.70 and 8.07 and the temperature between 16.1 °C and 29 °C. These are ideal physical conditions for the persistence of bacteriophages in the water.

Worldwide, 2.4 billion of people are without access to adequate sanitation facilities and a vast majority of individuals who are affected reside in developing countries [44]. Despite the lack of access to improved sanitation systems, most of the wastewater collected through the sewage systems in South Africa are inadequately treated and are discharged directly into rivers, lakes and the ocean [45]. This environmental water is used by individuals in many rural communities for drinking, recreational and agricultural purposes, and such direct use may pose health risks to humans [46]. In addition, such polluted environmental water also recharges groundwater sources. There is a general perception that groundwater is filtered by various layers of rocks, sand and soil and is free of chemical and biological impurities [47]. However, it was identified that groundwater is capable of transmitting gastroenteric pathogens and hepatitis A to humans [48]. In the present study, it was demonstrated that 15 of the 16 potable water samples from rural communities were positive for faecal indicator viral agents. In addition, previous studies have indicated that faecal indicator bacteria are present in groundwater sources of the North West Province [49,50,51,52,53]. The presence of bacteriophages in drinking water indicates a public health risk, and this amplifies the need to constantly assess the concentrations of viral pathogens in drinking water bodies and also evaluate the removal efficiency of the treatment processes [44].

Usually, the concentrations of bacteriophages in water decrease in winter and increase significantly throughout the warm summer months [54]. On the contrary, in this study, high numbers of somatic coliphages were obtained in winter than summer. F+RNA coliphages are of animal or human origin, unable to multiply in the general environment but are highly specific with regard to the infective site on the sex pillus, which makes them better indicators than somatic coliphages [55]. It is therefore suggested that somatic coliphages should be used concurrently with F+RNA coliphages when assessing water quality. The characterization of the F+RNA coliphages is an additional step that could assist in tracing pollution events [43] and contribute to the understanding and evaluating of the potential health risk associated with the consumption of untreated water.
